# Atg1 kinase organizes autophagosome formation by phosphorylating Atg9

**DOI:** 10.4161/auto.28971

**Published:** 2014-04-28

**Authors:** Daniel Papinski, Claudine Kraft

**Affiliations:** Max F Perutz Laboratories; University of Vienna; Vienna, Austria

**Keywords:** Atg1/ULK1 kinase, Atg18/WIPI2, Atg8, Atg9, Cvt pathway, autophagy

## Abstract

The conserved Ser/Thr kinase Atg1/ULK1 plays a crucial role in the regulation of autophagy. However, only very few Atg1 targets have been identified, impeding elucidation of the mechanisms by which Atg1 regulates autophagy. In our study, we determined the *Saccharomyces cerevisiae* Atg1 consensus phosphorylation sequence using a peptide array-based approach. Among proteins containing this sequence we identified Atg9, another essential component of the autophagic machinery. We showed that phosphorylation of Atg9 by Atg1 is required for phagophore elongation, shedding light on the mechanism by which Atg1 regulates early steps of autophagy.

The Ser/Thr kinase Atg1 is essential for the function of autophagy in yeast. Atg1 activity is regulated by nutrient-sensing pathways, making it a pivotal signal transducer in autophagy. Loss of Atg1 activity leads to aberrant localization of several other autophagy proteins and abrogates autophagosome formation, in line with a role of Atg1 kinase activity at an early step of autophagy.

Although the crucial role of Atg1 and its homologs from yeast to mammals has long been known and some kinase targets have been identified in higher eukaryotes, no target has been determined in yeast. To identify the first targets in *S. cerevisiae*, we employed a peptide array approach to determine the consensus phosphorylation sequence of Atg1. To ensure physiological specificity of the kinase, we affinity-purified genomically tagged Atg1 from *S. cerevisiae* with its native complex partners. We used the purified complex to in vitro phosphorylate randomized peptides containing a central serine or threonine phospho-acceptor site and one other fixed residue. We found that Atg1 strongly favored serine as the phospho-acceptor, in line with a recent publication by the Turk laboratory showing a link between the kinase active site sequence and acceptor specificity. Atg1 exhibited a distinct consensus sequence with strong selectivity for aliphatic residues at the -3 position and for aromatic and aliphatic residues at the +1 and +2 positions. Inversely, small, polar, and charged residues—determining the consensus of many other kinases—were strongly selected against at these positions.

We searched the *S. cerevisiae* proteome for sequences closely matching the Atg1 consensus and found more than 30 potential targets, including Atg1 itself and the autophagy factors Atg2 and Atg9. To check whether Atg1 can actually phosphorylate the candidate proteins, we subjected synthetic peptides spanning each consensus region to in vitro phosphorylation. More than 75% of the potential targets could be phosphorylated by Atg1 in vitro, highlighting the robustness of the determined Atg1 consensus sequence. While some targets phosphorylated in vitro may not be actual targets of Atg1 in vivo due to spatial restrictions, we could confirm Atg1-dependent phosphorylation of Atg1, Atg2 and Atg9 at Atg1 consensus sites in vivo using quantitative mass spectrometry, further validating the effectiveness of our consensus screen.

Atg9 is a multispanning transmembrane protein essential for autophagy. It localizes to cytoplasmic vesicles, a small number of which fuse at the phagophore assembly site (PAS) to nucleate autophagosome formation. Atg9 is retrieved from the forming autophagosome depending on Atg1 activity, as Atg9 accumulates at the PAS when Atg1 activity is abrogated. In vivo, we found Atg9 phosphorylation by Atg1 to be essential for autophagy. Expression of a nonphosphorylatable Atg9 serine-to-alanine mutant led to a near-complete block of both the Cvt pathway and bulk autophagy. This defect resulted from impairment of autophagy before autophagosome closure, as Atg9 phospho-mutants exhibited reduced protection of autophagic cargo from proteases. Interestingly, cellular localization of mutated Atg9 was indistinguishable from the wild-type protein. Thus, Atg1-dependent phosphorylation of Atg9 is dispensable for Atg9 transport to and from the PAS, and is not involved in Atg9 oligomerization, as nondimerizing Atg9 mutants show aberrant cellular localization.

Previous studies suggested that Atg9 is required to recruit Atg18 and Atg8 to the PAS. To assess whether phosphorylation of Atg9 triggers recruitment of these proteins, we analyzed their PAS localization. The number of puncta formed by either protein was reduced by more than 50% in an Atg9 phospho-mutant background. In the same background, the interaction of Atg18 with Atg9 was almost completely lost in co-immunoprecipitations, suggesting that Atg9 phosphorylation plays an active role in recruiting Atg18.

Finally, we analyzed the ability of live Atg9 phospho-mutant cells to enwrap autophagic cargo by fluorescence microscopy. Phagophores positive for GFP-Atg8 formed at and elongated around the cargo in the majority of wild-type cells; however, Atg9 mutants rarely formed phagophores and were unable to elongate them.

Atg9 is required for PAS recruitment of the PtdIns3K complex I, which by phosphorylating phosphatidylinositol provides binding sites for Atg18 on the phagophore. It is conceivable that Atg9 phosphorylation might trigger localization of the PtdIns3K complex I to the PAS by its autophagy-specific subunit Atg14 (Fig. 1). According to the genetic hierarchy proposed for PAS assembly, a defect in Atg14 recruitment would also explain the reduced puncta formation of Atg8 observed in the Atg9 phospho-mutant. However, we envision a more direct role of Atg9 phosphorylation in Atg18 function at the PAS. This is supported by the observation that while Atg18 PAS localization is not fully lost in the absence of Atg1-dependent Atg9 phosphorylation, autophagy is blocked completely. This coincides with a virtually complete loss of Atg18 binding to Atg9, suggesting that their interaction is required for autophagy function.

**Figure:**
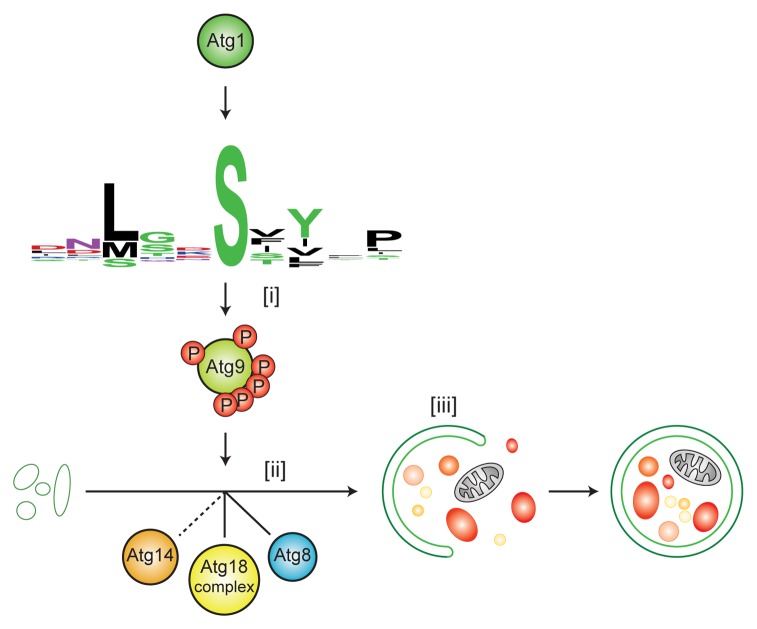
**Figure 1.** Atg1-dependent Atg9 phosphorylation regulates autophagy. Atg1 phosphorylates Atg9 on 6 consensus sites upon PAS recruitment (i). This allows the efficient recruitment of Atg18 and possibly Atg14 to the PAS (ii) and Atg9 binding to Atg18, which is required for Atg8 recruitment and phagophore elongation (iii) in autophagy and the Cvt pathway.

In summary, our work identifies the first Atg1 kinase target in budding yeast: Atg9. We found that in agreement with its role as the pivotal regulator of autophagy induction, Atg1 acts at an early stage of autophagy by directly phosphorylating Atg9 at multiple serine residues (Fig. 1). We showed that these phosphorylation events are required for efficient recruitment of Atg18 to the PAS and its interaction with Atg9, allowing subsequent Atg8 recruitment and phagophore elongation (Fig. 1). These findings provide a mechanistic explanation for the previously reported requirement of Atg1 kinase activity for phagophore elongation. As Atg9 is most likely not the only target of Atg1, future studies will help to elucidate the extent of signaling events mediated by this pivotal autophagy kinase.

## Notes

PapinskiDSchuschnigMReiterWWilhelmLBarnesCAMaiolicaAHansmannIPfaffenwimmerTKijanskaMStoffelI Early steps in autophagy depend on direct phosphorylation of Atg9 by the Atg1 kinase Mol Cell 2014 53 471 83 10.1016/j.molcel.2013.12.011

